# T4 Validation of the Burn Survivor Fear-avoidance Questionnaire

**DOI:** 10.1093/jbcr/irac012.003

**Published:** 2022-03-23

**Authors:** Bernadette Nedelec, Jocelyn Chen, Cassandra Caluori, Laura Alberton, Jinny Zhang, Danielle Shashoua, Valérie Calva, Nathalie Gauthier, Ana de Oliveira, Elisabeth Marois-Pagé

**Affiliations:** McGill University, Montreal, Quebec; McGill University, Montreal, Quebec; McGill University, Laval, Quebec; McGill, Montreal, Quebec; McGill University, Montreal, Quebec; Villa Medica Rehabilitation Hospital, Montreal, Quebec; Villa Medica Rehabilitation Hospital, Longueuil, Quebec; hôpital de réadaptation Villa Medica, St-Hubert, Quebec; CRCHUM, Montreal, Quebec; Hôpital de Réadaptation Villa Medica, Outremont, Quebec

## Abstract

**Introduction:**

According to the Fear-Avoidance (FA) Model, FA beliefs can lead to disability due to avoidance of activities expected to result in pain or further injury. Extensive research on the relationship of FA, pain, catastrophizing, and disability has been generated with patients suffering from chronic neck and back pain, but little research has been conducted with burn survivors. To address the need for a valid evaluation of FA in burn survivors, Langlois and colleagues developed, but did not validate, the Burn Survivor Fear-Avoidance Questionnaire (BSFAQ). Thus, the primary objective of this study was to investigate the construct validity of the BSFAQ among burn survivors. The secondary objective was to examine the relationship between FA and (i) pain intensity and (ii) catastrophizing at baseline (admission to rehab), 3 months and 6 months post-burn, and (iii) disability among burn survivors at 6 months post-burn.

**Methods:**

A prospective mixed methods approach was used to examine the construct validity by comparing the quantitative scores of the BSFAQ to independently performed qualitative interviews of burn survivors (n=31) that explored their lived-experiences, to determine if the BSFAQ discriminated those who had, from those who did not have FA beliefs and behaviors. Data for the secondary objective, scores of burn survivors (n=51) pain intensity (measured by the Numeric Rating Scale), catastrophizing (measured by the Pain Catastrophizing Scale), and disability (measured by the Burn Specific Health Scale-brief), were collected through a retrospective chart review.

**Results:**

For the primary objective, Wilcoxon Rank Sum Test results showed a statistically significant difference (p=0.015) between the BSFAQ scores of participants who were identified from the qualitative interviews as fear-avoidant compared to those who were identified as non-fear-avoidant. For the secondary objective, the Spearman correlation test results showed a moderate correlation between FA and (i) pain at baseline (r=0.466, p=0.002), a moderate correlation with (ii) catastrophizing thoughts over time (r=0.557, p=0.000; r=0.470, p=0.00; r=0.559, p=0.002 respectively at each time point), and a moderate correlation with (iii) disability at 6 months post-burn (r=-0.639, p=0.000).

**Conclusions:**

These results support that the BSFAQ is able to discriminate which BS are experiencing fear-avoidant beliefs and behaviours. As has been reported in other patient populations, burn survivors who express FA are more likely to report higher levels of pain early during their recovery that correlates with elevated catastrophizing thoughts, which are maintained across time and ultimately results in higher self-reported disability, which is consistent with the FA model.

